# Plasma and Tissue Concentrations of α-Tocopherol and δ-Tocopherol Following High Dose Dietary Supplementation in Mice

**DOI:** 10.3390/nu4060467

**Published:** 2012-06-06

**Authors:** Laura L. Baxter, Juan J. Marugan, Jingbo Xiao, Art Incao, John C. McKew, Wei Zheng, William J. Pavan

**Affiliations:** 1 Genetic Disease Research Branch, National Human Genome Research Institute, National Institutes of Health, Bethesda, MD 20892, USA; Email: lbaxter@mail.nih.gov (L.L.B.); aincao@mail.nih.gov (A.I.); bpavan@mail.nih.gov (W.J.P.); 2 Probe Development Branch, National Center for Advancing Translational Sciences, National Institutes of Health, Rockville, MD 20850, USA; Email: maruganj@mail.nih.gov (J.J.M.); xiaoj@mail.nih.gov (J.X.); 3 Therapeutic Development Branch, National Center for Advancing Translational Sciences, National Institutes of Health, Rockville, MD 20850, USA; Email: john.mckew@nih.gov

**Keywords:** vitamin E, α-tocopherol, δ-tocopherol, sesamin, pharmacokinetics

## Abstract

Vitamin E isoforms are essential nutrients that are widely used as dietary supplements and therapeutic agents for a variety of diseases. However, their pharmacokinetic (PK) properties remain poorly characterized, and high dosage animal studies may provide further information on their *in vivo* functions and pharmacological effects. In this study, alpha-tocopherol (α-toc) and delta-tocopherol (δ-toc) levels were measured in mouse plasma and tissues following their high dosage dietary supplementation. Average α-toc levels at 5, 10 and 20 g α-toc/kg diet increased over baseline levels 6-fold in plasma, 1.6-fold in brain, and 4.9-fold in liver. These elevated α-toc concentrations remained constant from 5 to 20 g α-toc/kg diet, rather than showing further increases across these dosages. No α-toc-related toxicity occurred at these high dosages, and strain-specific differences in liver and brain α-toc levels between Balb/cJ and C57Bl/6J mice were observed. Relatively high-dosage administration of dietary δ-toc for 1 or 4 weeks resulted in 6–30-fold increases in plasma and liver levels between dosages of 0.33 and 1.67 g δ-toc/kg diet. Co-administration of sesamin with δ-toc further increased δ-toc levels between 1.3- and 14-fold in plasma, liver, and brain. These results provide valuable PK information on high dosage α-toc and δ-toc in mouse and show that supplementation of sesamin with δ-toc further increases δ-toc levels over those seen with δ-toc supplementation alone.

## 1. Introduction

The molecules collectively known as vitamin E are fat-soluble compounds present in a wide variety of plant-derived foods. The 8 molecularly distinct isoforms of vitamin E are: alpha (α)-, beta (β)-, gamma (γ)-, and delta (δ)-tocopherol (toc), and α-, β-, γ-, and δ-tocotrienol (t3). The α-, β-, γ-, and δ- prefixes each identify the number and position of methyl groups on the aromatic chromanol ring. In addition, all the toc isoforms possess a saturated 15-carbon tail, while t3 tails are unsaturated [1]. The most prevalent vitamin E component in mammalian plasma and tissues is α-toc, and the other three tocs and t3s are found at much lower concentrations. For example, plasma of humans consuming a normal diet contains 22–34 µM α-toc, ≈10-fold less γ-toc and β-toc (4–5 µM), and ≈50-fold less δ-toc (0.3–0.8 µM) [2,3,4,5,6,7,8,9]. Each t3 is present at a 100-fold lower plasma concentration than α-toc (<1 µM) [1].

Vitamin E is an essential nutrient that was first identified 90 years ago based upon its effectiveness in countering fetal reabsorption in rats [10]. The necessity of vitamin E for human health is revealed in the detrimental phenotypes that occur in human diseases where vitamin E absorption or retention is severely limited, such as abetalipoproteinemia or ataxia with isolated vitamin E deficiency [11,12,13,14]. Interestingly, details are still emerging regarding the many functions that each vitamin E isoform can perform *in vivo*. *In vitro* studies show they are potent antioxidants, and also suggest they function in specific regulation of enzyme activity and gene expression, potentially stemming from roles in cell signaling and membrane fusion [1,15,16,17,18,19,20]. For example, the α-toc isoform has been reported to function as a pro-oxidant, as a modulator of gene transcription and enzymatic activity, and as an inhibitor of cell proliferation, platelet aggregation, and monocyte adhesion [15,20]. Additionally, the δ-toc isoform activates reporter gene transcription, inhibits cell proliferation, and selectively induces cell death in transformed cancer cell lines [21,22,23,24,25,26,27,28,29,30,31]. 

Alongside the continued discovery of various cellular activities affected by specific vitamin E isoforms, a growing body of data suggests that increased physiological toc/t3 levels in humans by altered dietary intake or supplementation might benefit a wide range of disease states. These include neurodegenerative disorders (Alzheimer’s disease, Parkinson’s disease, dementia), cystic fibrosis, amyotrophic lateral sclerosis, cancer, ischemia-reperfusion injuries, and temporal lobe radionecrosis [8,32,33,34,35,36,37,38,39,40]. These studies hint that vitamin E is not only needed to maintain normal physiological health, but may also delay or prevent disease if present at appropriate *in vivo* levels. However, even though the importance of sufficient vitamin E levels for normal human health is known from diseases of vitamin E deficiency, the effective levels of each vitamin E isoform for health benefits or disease prevention remain unclear. The interest in these potential human health benefits of vitamin E supplementation is reflected in the >25 clinical trials currently utilizing vitamin E [41]. 

Given the potential human health benefits of vitamin E supplementation, paired with knowledge gaps concerning vitamin E *in vivo* functions, a detailed understanding of the PK of each vitamin E isoform is needed. The most prevalent toc *in vivo*, α-toc, is widely used in synthetic vitamins and food supplements. Conversely, the least prevalent toc *in vivo*, δ-toc, has potent *in vitro* effects but little published *in vivo* data. Therefore, we performed *in vivo* experiments in mouse to identify PK profiles of α-toc and δ-toc. The α-toc levels in plasma, liver, and brain were measured in two mouse strains following 4 weeks of dietary supplementation with high dose α-toc. The PK profiles of single-dose δ-toc were determined, as well as its levels in plasma, liver, and brain following 1 week and 4 weeks of δ-toc dietary supplementation at relatively high doses as compared to the small amounts present in a typical diet. In addition, the sesame seed-derived lignan sesamin was co-administered with δ-toc, to determine if sesamin alters the PK of δ-toc, because sesamin has previously been shown to elevate plasma concentrations of α-toc, γ-toc, α-t3 in rat, and γ-toc and α-toc in humans [42,43,44,45,46,47]. The results of these studies revealed that high dosage dietary α-toc and δ-toc increased their respective concentrations in plasma, liver, and brain. Also, strain-specific differences in mean α-toc concentrations following high dosage dietary supplementation with α-toc were observed in liver and brain. Furthermore, co-supplementation of sesamin with δ-toc increased δ-toc concentrations throughout the body above concentrations achieved with δ-toc supplementation alone. 

## 2. Experimental Section

### 2.1. α-Toc, δ-Toc, Sesamin, and Mouse Diet

α-Toc (vegetable oil-derived) and δ-toc were obtained from Sigma-Aldrich (St. Louis, MO, USA) as technical grade (90% pure, catalog # T1539 and # T2028, respectively) and were subsequently purified by HPLC to >99% purity using supercritical fluid chromatography preparative systems at Lotus Separations, LLC (Princeton, NJ, USA). For α-toc purification, three separation methods were used in order to achieve the desired purity. In the first method, a Chiralpak IA (2 × 15 cm) column was used with an eluent of 15% isopropanol/CO_2_, 100 bar. Flow rate and collection wavelength was 60 mL/min and 220 nm, respectively. Injection volume was 0.4–0.7 mL at 30 mg/mL in methanol and isopropanol. In the second method, a Chiralpak IA (2 × 15 cm) column was used with an eluent of 20% isopropanol/CO_2_, 100 bar. Flow rate and collection wavelength was 85 mL/min and 220 nm, respectively. Injection volume was 0.75 mL at 30 mg/mL in methanol. In the third method, a Chiralpak AS-H (2 × 15 cm) column was used with an eluent of 18% methanol/CO_2_, 100 bar. Flow rate and collection wavelength was 75 mL/min and 220 nm, respectively. Injection volume was 1 mL at 30 mg/mL in methanol. For analysis, a Chiralpak AS-H (25 × 0.46 cm) column was used with an eluent of 20% methanol (DEA)/CO_2_, 100 bar. Flow rate was 3 mL/min and detection wavelengths were 220, 254, and 280 nm. Retention time was 1.95 min. For δ-toc purification, a Chiralpak AD-H (2 × 15 cm) column was used with an eluent of 10% ethanol/CO_2_, 100 bar. Flow rate and collection wavelength was 75 mL/min and 220 nm, respectively. Injection volume was 0.5 mL at 30 mg/mL in methanol. For analysis, a Chiralpak AD-H (25 × 0.46 cm) column was used with an eluent of 15% ethanol/CO_2_, 100 bar. Flow rate was 3 mL/min and detection wavelengths were 220, 280, and 295 nm. Retention time was 2.83 min.

Sesamin was obtained from Best PharmaTech, Inc. (Indianapolis, IN, USA). Mouse diet consisted of AIN93M as control diet, or AIN93M formulated with α-toc, δ-toc, or δ-toc + sesamin (Research Diets, Inc., New Brunswick, NJ, USA). The concentrations per kg of diet or per kg of body weight of α-toc, δ-toc, and sesamin are specified for each experiment. Of note, AIN93M contains 75 mg α-toc/kg and 900 µg vitamin K/kg [48].

### 2.2. Mice, α-Toc and δ-Toc Administration, and Tissue Collection

All animal work was done according to NIH-approved animal care and use protocols. A total of 20 BALB/cJ and 20 C57Bl/6J (Jackson Laboratories, Bar Harbor, ME, USA) male mice were used for high dosage α-toc supplementation in the diet. Mice of each strain were divided into 4 groups of 5 mice, and each group received one of the following diet formulations: Control, 5 g α-toc/kg diet, 10 g α-toc/kg diet, and 20 g α-toc/kg diet. Mice were fed each diet *ad libitum* for 4 weeks, then mice were euthanized and tissues collected.

A total of 30 male CD-1 mice were used for studies where single-dose δ-toc at 100 mg δ-toc/kg body weight was administered by intraperitoneal (IP) injection. For tissue collection, *N* = 3 at each timepoint. Plasma was collected at 0 min, 5 min, 15 min, 30 min, 1 h, 2 h, 4 h, 8 h, 12 h, and 24 h. Liver, kidney, lung, and brain were collected at 0 min, 4 h, 8 h, 12 h, and 24 h. 

A total of 231 male C57Bl/6 mice (SLAC Laboratory Animal Co. LTD, China) were used for studies in which single-dose δ-toc and sesamin in 100% corn oil were administered by oral gavage at a dose volume of 10 mL/kg. Of note, tocs are a natural component of corn oil, and thus small additional amounts of tocs were administered in each corn oil gavage at the following approximate concentrations (mg/kg body weight): α-toc = 0.002, β-toc = 0.00025, γ-toc = 0.004, δ-toc = 0.00025 [20,49]. Mice were divided into 7 groups of 33 mice, and each group was given a single dose of one of the following δ-toc/sesamin concentrations (all in mg/kg body weight): (1) 240 mg sesamin; (2) 10 mg δ-toc; (3) 50 mg δ-toc; (4) 250 mg δ-toc; (5) 240 mg sesamin + 10 mg δ-toc; (6) 240 mg sesamin + 50 mg δ-toc; and (7) 240 mg sesamin + 250 mg δ-toc. For tissue collection, *N* = 3 per group at each of 11 timepoints: 0 min, 5 min, 15 min, 30 min, 1 h, 2 h, 4 h, 8 h, 12 h, 24 h, and 48 h. 

A total of 60 Balb/cJ mice (Jackson Laboratories) were used for high dosage dietary supplementation of δ-toc and δ-toc + sesamin, with 30 mice each for the 1-week and 4-week studies. In all cases of sesamin supplementation, concentration was 2 g sesamin/kg diet. Six groups of 5 mice were each given diets supplemented with one of the following δ-toc/sesamin concentrations (all in mg/kg diet): (1) Control (AIN93M), (2) 0.33 g δ-toc, (3) 1.67 g δ-toc, (4) sesamin + 0.067 g δ-toc, (5) sesamin + 0.33 g δ-toc, and (6) sesamin + 1.67 g δ-toc.

For serum collection, blood samples were collected into chilled, heparinized tubes by retro-orbital sinus bleed or terminal cardiac puncture. After cooling to 4 °C, blood samples were centrifuged to separate serum, and isolated serum was snap frozen at −80 °C. For all other tissue collections, mice were euthanized, and subsequently tissues were collected and immediately snap frozen at −80 °C.

### 2.3. Liquid Chromatography-Mass Spectrometry

Analytical samples were prepared by homogenizing tissue in 3 volumes of 1-propanol per gram of tissue, then centrifuging to remove precipitated proteins. Samples were analyzed using an Agilent 6410 mass spectrometer coupled with an Agilent 1200 HPLC and a CTC PAL chilled autosampler, all regulated by MassHunter software (Agilent). After separation on a C_18_ reverse phase HPLC column (Agilent, Waters, or equivalent) using a methanol-water gradient system, peaks were analyzed by mass spectrometry using electrospray ionization in multiple reaction monitoring mode. Mobile phase A was 7.5 mM ammonium acetate in water, mobile phase B was 0.1% formic acid in methanol, and the flow rate was 1 mL/min. The gradient program included a 0.5 min hold at 20% B (the starting conditions), followed by a gradient to 99% B over 1.25 min and a 1 min hold at 99% B. The column was then returned to starting conditions and equilibrated over 1.25 min. Calibration curves for each tissue were determined by preparing a 50× stock of α-toc or δ-toc in 1-propanol, then generating serial dilutions. These serial dilution samples were then diluted 50-fold into homogenized tissues.

### 2.4. Data Analysis

One-way or two-way ANOVA tests with post-hoc Tukey-Kramer multiple comparisons tests were performed to compare mean α-toc and δ-toc levels, with *p* < 0.05 deemed significant. Values below the lower limit of quantitation (<LLOQ) were estimated at LLOQ/2 [50]. Area under concentration-time curves (AUCs) from 0–24 (AUC_0–24_) or 0 to 48 h (AUC_0–48_) were determined using the trapezoidal rule, and mean AUCs were compared by two-tailed *t*-tests.

## 3. Results

### 3.1. α-Toc

**Figure 1:**
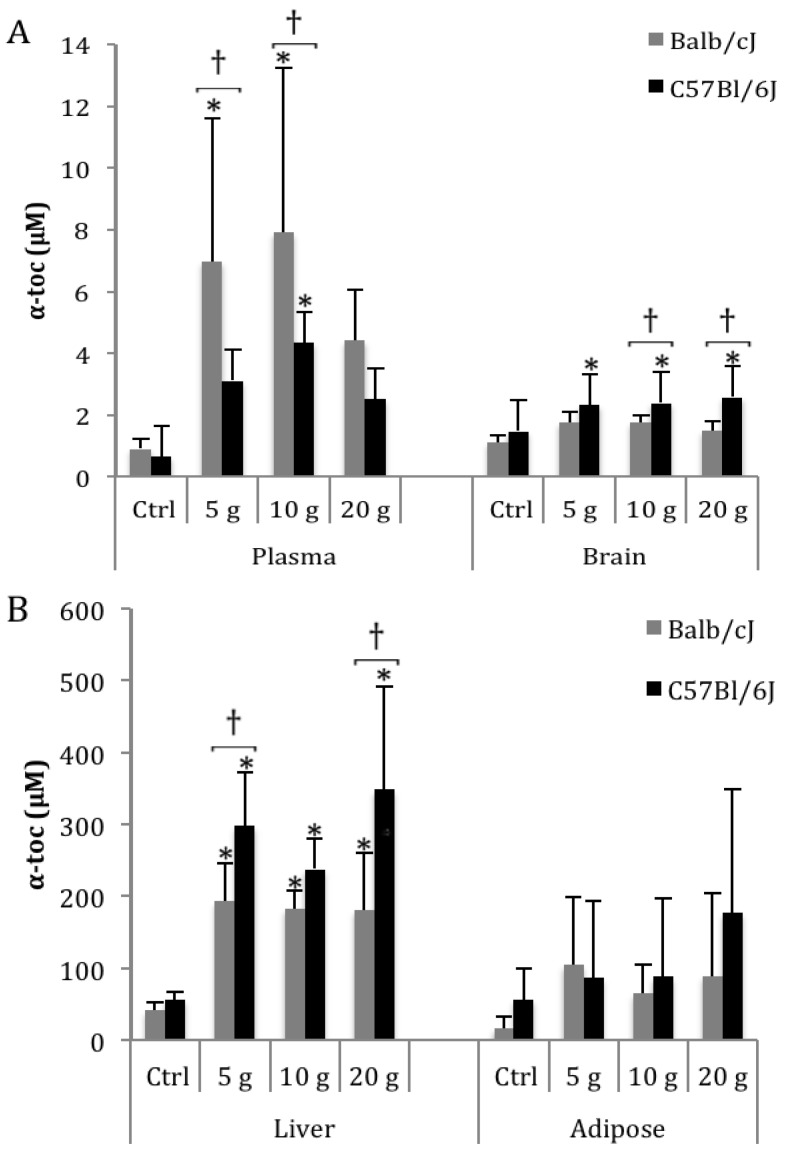
Mean plasma, brain, liver, and adipose α-toc levels following 4 weeks high dosage α-toc dietary administration to adult Balb/cJ and C57Bl/6J mice. Supplementation at 5, 10, or 20 g α-toc/kg diet resulted in significantly different α-toc levels in plasma (**A**), brain (**A**), and liver (**B**) by one- or two-way ANOVA (*p* < 0.05); Tukey-Kramer post-hoc tests showed significant differences from control in some tissues (indicated by *, *p* < 0.05), most notably in liver and C57Bl/6J brain. Significant strain-specific differences in α-toc levels in Balb/cJ and C57Bl/6J mice were apparent by two-way ANOVA (*p* ≤ 0.009); Tukey-Kramer post-hoc tests showed significant strain-specific differences at some dosages in plasma, brain and liver (indicated by †, *p* ≤ 0.05). Adipose (**B**) α-toc levels showed no significant differences. No significant differences were seen among mean α-toc levels at the 5, 10, and 20 g α-toc doses in plasma or tissues. *N* = 5 mice/strain/dosage; error bars = positive standard deviation.

Adult Balb/cJ and C57Bl/6J mice were given high dosage dietary α-toc for 4 weeks, at concentrations of 0 (control), 5, 10 and 20 g α-toc /kg diet. Subsequently, the mice were examined for pathological abnormalities, and the α-toc concentrations in plasma, brain, liver, and adipose tissue were measured (Figure 1, Supplementary Table S1). Pathological analyses of liver, brain, kidney, heart, and skeletal muscle all showed no notable abnormalities that differed from controls (Supplementary Table S2), suggesting no toxicity from high dose α-toc ingestion in these mice. Dietary supplementation with α-toc resulted in increased α-toc levels in plasma, brain, and liver, with significantly different mean α-toc levels occurring in these 3 tissues (one-way ANOVA, *p* < 0.05 for each strain). In contrast, adipose α-toc levels showed no significant differences from controls for either strain at any dosage (Figure 1B). Post-hoc analyses showed significant differences between mean α-toc levels in control and supplemented mice at all liver dosages, at all dosages in C57Bl/6J brain, and in plasma at 5 and 10 g α-toc dosages (indicated by * in Figure 1, *p* < 0.05). Modest α-toc increases of less than 2-fold were seen in brain (*i.e.*, from 1.1 µM to 1.49–1.75 µM in Balb/cJ, Figure 1A), while larger increases of 4–9-fold were observed in plasma (*i.e.*, from 0.9 µM to 4.4–7.9 µM in Balb/cJ, Figure 1A) and liver (*i.e.*, from 40.6 µM to 182–193 µM in Balb/cJ, Figure 1B). Notably, mean plasma, brain, and liver α-toc levels within each mouse strain at 5, 10 and 20 g α-toc/kg diet showed no significant differences from each other (one-way ANOVA, *p* ≥ 0.2). These data indicated that α-toc concentrations remained constant in these tissues even though dietary dose escalated from 5 to 20 g α-toc/kg diet, and suggested the existence of an *in vivo* α-toc concentration plateau at or below 5 g α-toc/kg diet. 

Interestingly, two-way ANOVA revealed significantly different mean plasma, liver, and brain α-toc concentrations when comparing not only α-toc dosage groups, but also mouse strains (for α-toc dosage, *p* ≤ 0.002; for mouse strain, *p* ≤ 0.009). Mean plasma α-toc concentrations were higher in Balb/cJ mice, while brain and liver α-toc concentrations were higher in C57Bl/6J mice. Post-hoc analyses comparing these two mouse strains revealed statistically significant differences in mean α-toc concentrations at some dosages (indicated by † in Figure 1, *p* < 0.05). Taken together, these results indicate that α-toc concentrations increase in mouse plasma, brain, and liver following dietary supplementation of large dosage α-toc, and these α-toc concentrations in these tissues plateau at a dosage at or below 5 g α-toc/kg diet. In addition, C57Bl/6J and Balb/cJ mouse strains show quantitatively different *in vivo* α-toc levels following α-toc supplementation, suggesting allelic variation between these strains might contribute to altered efficiency of the metabolic pathways regulating α-toc storage and removal.

### 3.2. δ-Toc

**Figure 2:**
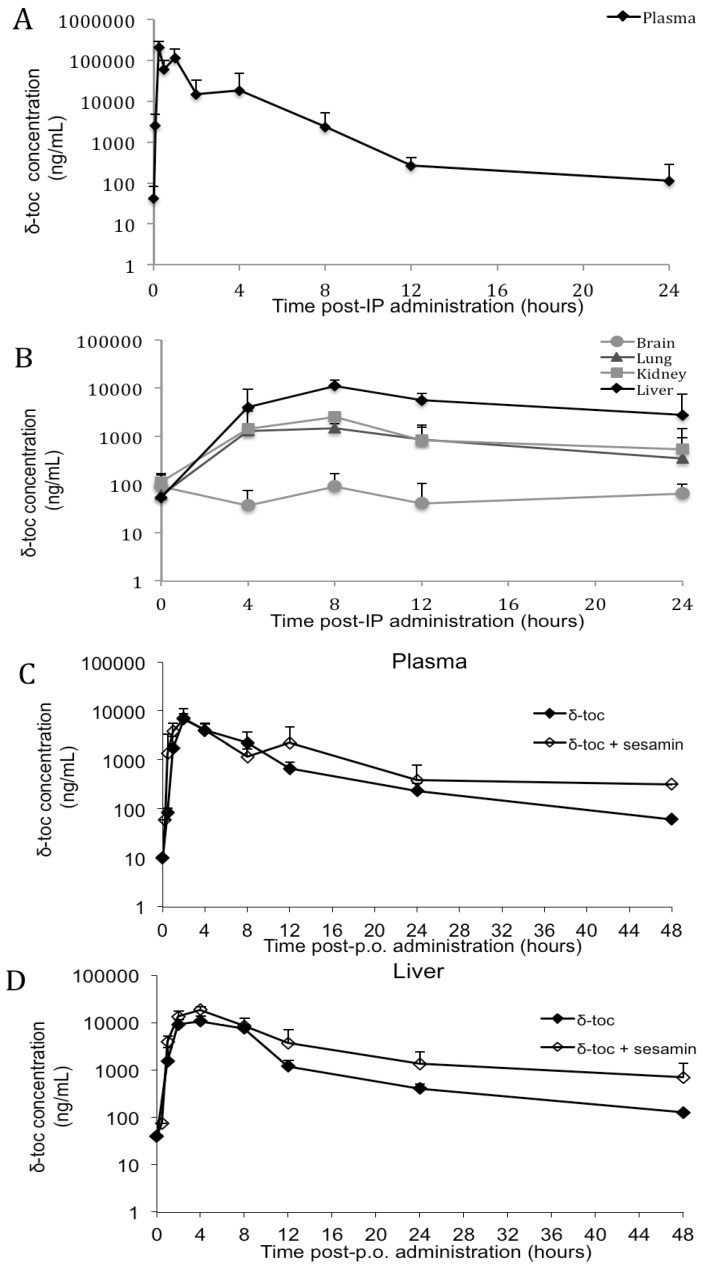
Concentration-time profiles of δ-toc following single dosage administration of δ-toc or δ-toc with sesamin by IP or p.o. routes. (**A**,**B**) Following single dosage IP δ-toc at 100 mg/kg body weight, mean plasma (**A**) and mean liver, kidney, lung, and brain δ-toc concentrations (**B**) were measured at the indicated timepoints. (**C**,**D**) Following single p.o. dosage of either δ-toc alone at 250 mg/kg body weight (filled diamonds) or δ-toc + sesamin at 250 and 240 mg/kg body weight, respectively (open diamonds), average plasma (**C**) and liver (**D**) δ-toc concentrations were measured at the indicated timepoints. The δ-toc concentrations are plotted on a log scale, and error bars show positive standard deviation. *N* = 3 mice/timepoint/dosage.

There is very little published PK data describing either single or long-term dosages of δ-toc. Thus, in order to generate PK data on single dosage δ-toc in mouse, adult mice were given single IP injections of δ-toc at 100 mg/kg body weight, and plasma, brain, lung, kidney, and liver concentrations of δ-toc were measured at timepoints spanning 0 to 24 h. The mean plasma *T*_max_ for δ-toc was 15 min, and lung, kidney and liver each had a mean *T*_max_ of 8 h (Figure 2A,B). The mean plasma AUC_0–24_ was 241 h·µg/mL. Over the timecourse, δ-toc levels decreased but did not reach baseline by 24 h, with greatest δ-toc retention seen in liver. Brain δ-toc levels remained low and unchanged throughout the timecourse. 

Next, single dosage δ-toc was administered to adult mice by oral gavage (p.o.) at 10, 50, or 250 mg/kg body weight. Subsequently, plasma, liver, and brain concentrations of δ-toc were measured at timepoints from 0 to 48 h (Figure 2C,D for 250 mg/kg dosage; Supplementary Figure S1 for 10 and 50 mg/kg dosages). The δ-toc *T*_max_ in plasma and liver was 2 or 4 h at all dosages. By 24 h, δ-toc levels in plasma and liver had returned to baseline at the 10 mg/kg dosage. At the 50 mg/kg and 250 mg/kg dosages, the δ-toc levels rapidly decreased but did not reach baseline by 48 h. Brain δ-toc levels remained undetectable at all dosages and timepoints. 

Sesamin, a component of sesame seeds, has been reported to increase plasma and tissue concentrations of α-toc, γ-toc, α-t3, and γ-t3 by inhibiting their metabolism [42,44,45,46,47,51,52]. To investigate whether δ-toc plasma and tissue concentrations can be increased by sesamin, single p.o. doses of 10, 50, or 250 mg δ-toc /kg body weight were administered along with sesamin at 240 mg/kg body weight, and subsequent plasma, liver, and brain δ-toc levels were measured at timepoints from 0–48 h (Figure 2C,D for 250 mg/kg dosage; Supplementary Figure S1 for 10 and 50 mg/kg dosages). Similar to what was seen for p.o. δ-toc alone, p.o. δ-toc with sesamin gave *T*_max_ values of 2 to 4 h in plasma and liver, δ-toc concentrations either reached baseline or neared baseline by 24 to 48 h, and brain δ-toc levels remained undetectable. Interestingly, comparison of the mean AUC_0–48_ values for 250 mg δ-toc alone with those for 250 mg δ-toc with sesamin revealed that coadministration of sesamin did not alter δ-toc concentration in plasma (δ-toc alone = 43.0 h·µg/mL; δ-toc + sesamin = 56.1 h·µg/mL; *p* = 0.44), but significantly increased liver δ-toc concentration (δ-toc alone = 95.1 h·µg/mL; δ-toc + sesamin = 173 h·µg/mL; *p* = 0.02). These data suggest co-administration of sesamin with δ-toc has the potential to increase *in vivo* δ-toc concentrations.

To further investigate sesamin’s effect on δ-toc levels, and to generate PK data for long-term δ-toc supplementation, plasma and tissue δ-toc concentrations were determined following long-term dietary supplementation with high dosage δ-toc and high dosage δ-toc with sesamin. Balb/cJ mice were exclusively fed chow containing high dosage δ-toc (at 0.33 or 1.67 g/kg diet) or high dosage δ-toc with sesamin (0.067, 0.33, or 1.67 g δ-toc/kg diet + 2 g sesamin/kg diet) for 1 week or 4 weeks. Pathological analyses of livers from mice receiving long-term dietary supplementation of δ-toc or δ-toc with sesamin showed no notable abnormalities that differed from those of controls (Supplementary Table S3), indicating no toxicity from high dose δ-toc ingestion.

After 1 week (Table 1), significant changes in mean δ-toc levels occurred that were attributable to both supplemental δ-toc dosages and sesamin co-supplementation (by two-way ANOVA), with *p* ≤ 0.0004 for δ-toc supplementation in plasma, brain, and liver, and with *p* ≤ 0.02 for sesamin co-supplementation in brain and liver. Similarly, 4 weeks of supplemental high dosage δ-toc alone or with sesamin (Table 2) caused significant changes in mean plasma and liver δ-toc levels (by two-way ANOVA), with *p* ≤ 0.00002 for δ-toc supplementation in plasma and liver, and *p* ≤ 0.04 for sesamin co-supplementation in plasma and liver (brain was not analyzed due to <LLOQ levels for 4 out of 5 dosages in the 4 week studies). 

**Table 1:** Concentration (µM) of δ-toc in plasma, brain, and liver of Balb/cJ mice following 1 week dietary supplementation with δ-toc or δ-toc plus sesamin ^1^.

Diet (g/kg diet)	1 week		
	Plasma ^2^	Brain	Liver
Control	<LLOQ	0.030 (0.017) ^a^	<LLOQ
0.33 δ-toc	0.080 (0.053) ^a^	0.036 (0.023) ^a^	0.150 (0.114) ^a^
1.67 δ-toc	0.777 (0.300) ^b^	0.053 (0.013) ^a^	4.618 (2.163) ^b^
0.067 δ-toc + 2 sesamin	0.032 (0.019) ^a^	0.032 (0.016) ^a^	0.394 (0.736) ^a^
0.33 δ-toc + 2 sesamin	0.258 (0.150) ^a^	0.039 (0.023) ^a^	2.130 (1.443) ^a,b^
1.67 δ-toc + 2 sesamin	0.995 (0.388) ^b^	0.135 (0.055) ^b^	9.753 (2.627) ^c^

^1^ Standard deviation in parentheses. ^2^ Within each column, δ-toc concentrations without a shared letter (^a,b,c^) are significantly different by ANOVA with Tukey-Kramer multiple comparisons test, *p* < 0.05.

**Table 2:** Concentration (µM) of δ-toc in plasma, brain, and liver of Balb/cJ mice following 4 weeks dietary supplementation with δ-toc or δ-toc plus sesamin ^1^.

Diet (g/kg diet)	4 weeks		
	Plasma ^2^	Brain	Liver
Control	<LLOQ	<LLOQ	1.330 (1.078) ^a^
0.33 δ-toc	0.0132 (0.006) ^a^	<LLOQ	2.064 (0.254) ^a^
1.67 δ-toc	0.324 (0.232) ^a^	<LLOQ	12.71 (6.571) ^a^
0.067 δ-toc + 2 sesamin	0.015 (0.009) ^a^	<LLOQ	2.547 (1.022) ^a^
0.33 δ-toc + 2 sesamin	0.130 (0.116) ^a^	<LLOQ	6.189 (5.114) ^a^
1.67 δ-toc + 2 sesamin	0.947 (0.411) ^b^	0.259 (0.039)	25.60 (12.40) ^b^

^1^ Standard deviation in parentheses. ^2^ Within each column, δ-toc concentrations without a shared letter (^a,b,c^) are significantly different by ANOVA with Tukey-Kramer multiple comparisons test, *p* < 0.05.

**Figure 3:**
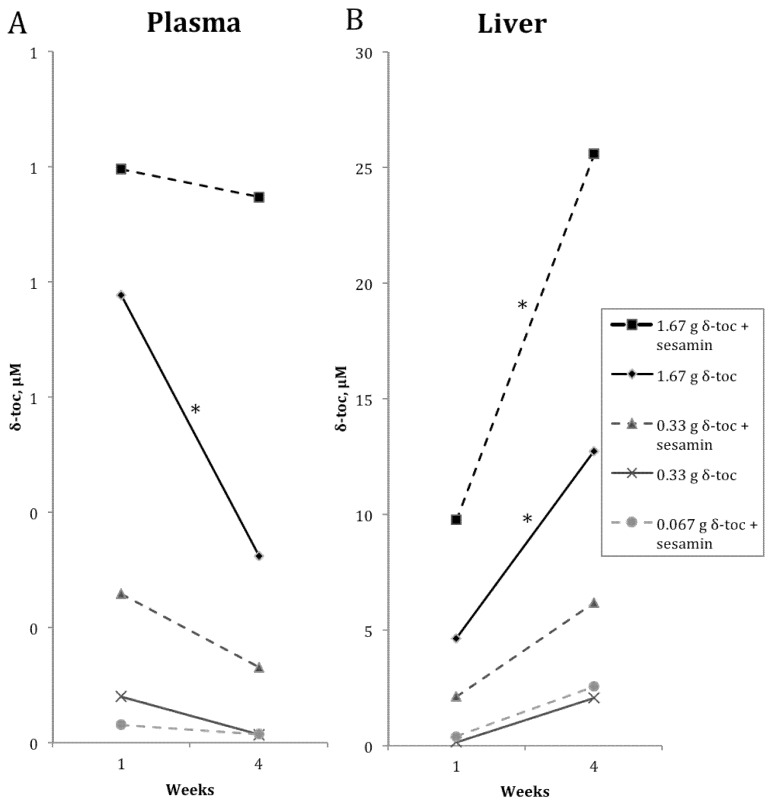
Comparison of mean δ-toc concentrations between the 1- and 4-week supplemental dietary δ-toc groups. (**A**) Plasma δ-toc levels decreased between 1 and 4 weekswhen supplemental δ-toc at 1.67 g δ-toc/kg diet was given alone, but the co-supplementation of sesamin with δ-toc prevented this decrease. (**B**) Liver δ-toc levels increased between 1 and 4 weeks when supplemental δ-toc at 1.67 g δ-toc/kg diet was given alone, and co-supplementation of sesamin with δ-toc achieved higher δ-toc levels. No differences were seen at any of the 0.067 g or 0.33 g δ-toc dosages when comparing the 1-week and 4-week groups. * Statistically significant difference between mean δ-toc concentrations at 1 and 4 weeks by ANOVA followed by Tukey-Kramer multiple comparisons test, *p* < 0.05.

Comparison of δ-toc concentrations between the 1- and 4-week supplemental dietary δ-toc groups (Table 1 *vs.*Table 2) revealed significant differences in mean δ-toc levels by two-way ANOVA in plasma (*p* = 0.03) and liver (*p* = 0.00004). While post-hoc analyses showed no differences at the 0.067 g or 0.33 g δ-toc dosages between the two time periods, either with or without sesamin, significant changes were seen at the 1.67 g δ-toc dosages (Figure 3). When 1.67 g δ-toc alone was given, plasma levels significantly decreased while liver levels increased between the 1 and 4 week time periods. These results suggest tissue-specific and time-dependent δ-toc distribution, with δ-toc being more efficiently removed from blood and increasingly retained in liver over time. Furthermore, the co-administration of sesamin with the 1.67 g δ-toc dosage increased liver d-toc levels, increased brain δ-toc levels (from 0.135 µM at 1 week to 0.259 µM at 4 weeks, *p* = 0.01), and also maintained plasma δ-toc levels. These data suggest that over long time periods of administration, sesamin can allow increased *in vivo* δ-toc levels, by limiting the removal of circulating δ-toc and also by increasing accumulation of δ-toc in tissues such as liver and brain. 

Additional post-hoc analyses comparing mean δ-toc concentrations at each dosage within plasma, brain, or liver showed that only a subset of δ-toc levels significantly differed from one another (Table 1 and Table 2, significance indicated by different superscripts ^a,b,c^). In all tissues, only the 1.67 g δ-toc dosages achieved significant increases in δ-toc concentrations. In regards to sesamin co-administration, only at the 1.67 g dosages did co-administration of sesamin cause a significant increase over the levels achieved for δ-toc alone (at 4 weeks in plasma, in liver at 1 and 4 weeks, and in brain at 1 week). Similarly, high brain δ-toc levels were achieved when sesamin was administered with the 1.67 g δ-toc dose for 4 weeks (0.285 µM). Taken together, these data suggest measurable effects by sesamin on δ-toc concentration cannot be achieved at low dosages, on the order of 0.067–0.33 g/kg diet, but instead require higher δ-toc dosages, on the order of 1.67 g δ-toc/kg diet. 

## 4. Discussion

Although the antioxidant effect of vitamin E has been extensively reported, the extent of its *in vivo* biological effects remains unclear. The therapeutic effects of vitamin E on a wide range of diseases continue to be explored both in experimental animals and in human clinical trials. Understanding of vitamin E PK profiles in experimental species is critical to guide experimental design and selection of correct vitamin E dosages. To date, some data have been reported regarding basal plasma and tissue levels of vitamin E isoforms and their levels following low dosage supplementation. For example, the basal plasma concentration of α-toc in humans is 22–34 μM, increasing to 56–70 μM when 671 mg/day of α-toc is supplemented [53]. In mice fed standard diets, the basal plasma concentration of α-toc is 5.0–20 μM, increasing to 19–45 µM at 1–3 g/kg diet [54,55,56,57]. In both humans and rodents, liver α-toc concentrations are consistently higher than those of plasma [55,58,59,60]. However, the PK profiles for many vitamin E isoforms, especially when administered in large doses, are not completely available in mouse. This study has filled some of the knowledge gaps that existed for PK profiles of high dosage α-toc and δ-toc in mice. Also, this study demonstrated that co-administration of sesamin altered the PK profile of high dosage δ-toc, resulting in increased δ-toc concentrations.

### 4.1. α-Toc

High dosage dietary supplementation of α-toc significantly increased α-toc levels over baseline in Balb/cJ and C57Bl/6J mice, however these increased α-toc concentrations remained constant above dosages of 5 g α-toc/kg diet. Of note, this apparent *in vivo* plateau may extend to α-toc dosages that are lower than 5 g α-toc/kg diet, and in fact other studies using different mouse strains achieved slightly higher plasma concentrations at 1–3 g α-toc/kg diet [53,55]. Future studies analyzing lower α-toc dosages in a variety of mouse strains could precisely define the minimal concentration in each strain that is sufficient to achieve consistently high *in vivo* α-toc concentrations. The occurrence of relatively constant *in vivo* α-toc concentrations in spite of increased α-toc intake may simply reflect the presence of an upper limit to intestinal absorption of α-toc [61]. However, this also may suggest saturation paired with escalating removal of α-toc in response to high dosage ingestion, a hypothesis supported by the gradual decrease in peak α-toc levels over time when high doses of α-toc are administered subcutaneously in rat [62]. This study goes on to demonstrate long-term high dose α-toc results in the upregulation of proteins responsible for α-toc metabolism or removal, including cytochrome P450 (CYP) enzyme CYP3A and multidrug resistance protein 1 (MDR1) [62]. An additional study in mouse demonstrates that high dosage α-toc results in upregulation of CYP3A11, the mouse ortholog of CYP3A [63]. 

No acute toxic effects were seen in mouse as a result of 4 weeks of high dosage α-toc. However, these mice did not harbor any diseases or developmental genetic abnormalities, nor were they subjected to any physiological or environmental stressors. Therefore future studies are warranted to determine if α-toc metabolism differs under these types of challenges such that detrimental effects from high dosage α-toc could occur. In general, tocs/t3s are expected to have low toxicity because of their rapid metabolism by CYP enzymes in the liver, a characteristic that contrasts with the toxic accumulation seen from high dosage ingestion of other fat-soluble vitamins. Accordingly, extremely high dosage α-toc (2.5–10 g α-toc/day) can be administered to human patients with rare disorders that cause vitamin E deficiency, as well as to patients with amyotrophic lateral sclerosis [11,13,14,33,64,65]. These high dosages are not reported to be toxic even when administered long term, provided supplemental vitamin K is also given to prevent vitamin E antagonizing vitamin K-mediated blood coagulation [53]. However, toxic effects were reported in early studies where high dosage α-toc alone was administered, including hemorrhagic toxicity in rat and chick at dosages of 1–2 g α-toc/kg body weight [66,67]. The rationale behind these different results regarding toxicity of high dosage α-toc in mouse, human, rat, and chicken is unclear, and would be clarified by additional future quantitative studies of high dosage α-toc in each model organism, as well as clinical data from humans taking more modest supplemental levels.

Mouse brain showed small increases in α-toc levels in response to high dosage α-toc supplementation when compared to those of plasma and liver. On average, the brain showed a 1.6-fold increase to 2 µM, as compared to the 6-fold increase in plasma to 4.9 µM and the 4.9-fold increase in liver to 240 µM. This suggests the presence of brain-specific mechanisms that limit α-toc uptake into the brain, potentially at the blood-brain barrier. An understanding of the brain’s response to elevated intake of tocs is important for interpretation of neurological studies, which have suggested elevated dietary intake and plasma levels of tocs/t3s may play a role in delaying neurological disease in humans and mouse models [8,38,39,68,69,70,71,72]. Some human studies have shown that high dosage α-toc slows disease progression in patients with Alzheimer’s disease and Parkinson’s disease [36,73]. However, other studies showed no benefit from α-toc supplementation in patients with neurological disease [74,75,76,77], and some even suggested risks to healthy individuals from α-toc supplementation, reporting an increased risk of mortality [78] or hemorrhagic stroke [79,80]. Clearly our understanding of the effects of α-toc supplementation in humans remains limited and requires further study. Additionally, animal model studies are needed to determine if baseline or high dosage *in vivo* α-toc levels differ between normal animals and those exhibiting various diseases.

### 4.2. δ-Toc

This study revealed that the oral administration of high dosage δ-toc increased δ-toc levels throughout the body, and these δ-toc levels further increased with co-administration of sesamin at the highest dosage of 1.67 mg δ-toc/kg diet. To our knowledge, this is the first report showing sesamin can increase *in vivo* δ-toc levels in mouse plasma, liver, and brain. Sesamin is a naturally occurring component of sesame seeds, and functions *in vivo* as an antioxidant [81], anti-cancer agent [82,83,84], anti-hypertension agent [85,86,87,88,89,90,91] and reducer of serum lipids [91,92,93,94,95,96]. Functional analyses of sesamin *in vitro* have shown that it blocks CYP oxidation/excretion of tocs and t3s [97,98], thus suggesting a mechanism whereby sesamin maintains higher plasma toc levels. This function has in fact been demonstrated in rat, where sesamin inhibits γ-toc breakdown and elevates plasma concentrations of α-toc, γ-toc, and α-t3 [42,43,44,45], and also in human, where sesamin elevates plasma concentrations of γ-toc and α-toc [46,47]. However, sesamin does not have uniform effects on all tocs/t3s, showing more frequent effects on γ-toc than α-toc, and even reducing β-toc levels [42,46,47]. Thus independent analyses of the *in vivo* effect of sesamin on each toc/t3 are required.

The brain exhibited lower δ-toc concentrations than those in plasma and liver, similar to the lower brain α-toc concentrations seen following α-toc supplementation. Therefore it appears that the ability to elevate δ-toc levels in the brain and the resulting potential to alleviate phenotypes of neurological disease is severely restricted. However, it remains possible that, even at low concentrations, the unique chemical properties of δ-toc based upon its structure may result in beneficial physiological effects in some parts of the body. For example, some animal studies suggest δ-toc may display properties that can be used to prevent cancer progression [99]. Since each toc varies in the number and position of methyl groups on the aromatic chromanol ring, each has a different antioxidant potential, via hydrogen donation from methyl groups, or electrophile trapping ability, via aromatic substitution at free ring positions. The single methyl group and two free aromatic ring positions of δ-toc predict that δ-toc has lower oxidation potential but higher electrophile scavenging potential. Future studies are needed to discover the cellular functions allowed by the unique structure of δ-toc, whether these effects are beneficial to disease, and if so, the optimal physiological concentrations to allow δ-toc to exert these beneficial effects. Also, future studies will need to determine if the *in vivo* δ-toc levels achieved following high dosage ingestion are different between normal and disease states.

In these studies, δ-toc plasma and tissue concentrations never reached levels that were similar to those of α-toc, even when co-administered with sesamin. While this suggests that mouse may not be a tractable animal model for studying the *in vivo* functions of δ-toc, it also suggests that high dosage of δ-toc or δ-toc with sesamin did not radically change the rate of normal δ-toc metabolism. High concentrations of α-toc are maintained by the preferential binding and release of α-toc to the plasma by α-toc transfer protein (α-TTP), while lower concentrations of the other seven vitamin E isoforms result from their rapid, CYP-mediated breakdown [97,100,101,102,103]. The biological significance of this selective α-toc enrichment over the other tocs/t3s *in vivo* is unknown, although functional importance is supported by the conservation of α-toc enrichment across vertebrates [104]. It does not simply reflect a prevalence of α-toc in dietary sources, as plant oils that are highly consumed in the Western diet (peanut, soybean) contain greater amounts of γ-toc than α-toc [1]. However, toc ingestion can affect toc concentration to some degree, as increased dietary intake of individual toc/t3 isoforms directly correlated with their increased plasma and tissue levels in humans and animal models ([54,105,106,107,108,109,110] and this study). Interestingly, dietary toc supplementation of one toc has been shown to perturb the *in vivo* levels of other tocs. Supplemental α-toc decreased levels of δ-toc and γ-toc [5,111,112,113,114,115], and conversely, γ-toc supplementation decreased α-toc levels [54,110]. This added complexity indicates that it is necessary to understand not only the PK of each vitamin E isoform but also their effects on each other’s concentration and function *in vivo*. This will allow correlation of the different biological functions of each isoform, often determined from *in vitro* studies, with their potential for *in vivo* therapeutic applications. The efficient achievement of therapeutic levels may require adjustment of the *in vivo* ratios of individual vitamin E isoforms. 

### 4.3. Toc Levels and Strain-Specific Variation

The baseline α-toc and δ-toc levels reported here correlate with a subset of previous mouse studies. In mice fed standard diets, previously reported plasma levels were 5.0–20 µM for α-toc [54,55,56,57] and 0.08 µM for δ-toc [54]. Organ levels of α-toc have been previously reported as 20–27 µM in liver, 64 µM in adipose, and 5–50 µM in brain [55,57,60,116]. These previously reported basal concentrations of liver α-toc and adipose α-toc are similar to those in this study, however those of plasma δ-toc, plasma α-toc, and brain α-toc exceed those in this study.

Similarly, the α-toc and δ-toc levels reported in this study resulting from high dosage α-toc and δ-toc showed both similarities and differences to previous mouse studies. Supplemental α-toc at 5 g α-toc/kg diet resulted in liver concentrations similar to those found in our study (156–207 µM), but greater α-toc concentrations in brain (25–29 µM) [55]. Supplementation of α-toc at dosages ranging from 1–3 g α-toc/kg diet resulted in higher plasma α-toc concentrations (19–45 µM) than those of this study [54,56]. Plasma levels resulting from supplemental δ-toc at 0.3% of diet correlated with the δ-toc plasma levels obtained in this study (0.5–1.2 µM) [54]. 

The higher brain and plasma levels in these previous studies could reflect the usage of longer time periods of diet supplementation (8–48 weeks *vs.* 4 weeks in current study), or may reflect strain-specific variation affecting toc metabolism, as these studies used a variety of mouse strains. The strain-specific differences in α-toc levels between Balb/cJ and C57Bl/6J identified in the current study support the latter hypothesis, as does the existence of human polymorphisms that are associated with functional differences in vitamin E metabolism. These include missense mutations in the toc/t3 metabolic enzyme CYP4F2 that result in differing enzymatic activity [117], as well as polymorphisms in lipoprotein transport proteins that are associated with variable plasma α-toc levels [7,118,119,120,121,122,123]. Further studies are needed to discover similar polymorphisms in inbred mouse strains and to determine how they contribute to variable vitamin E metabolism [124]. 

## 5. Conclusions

This study provides essential quantitative and strain-specific PK information on administration of high dosage α-toc and δ-toc in the mouse. High dosage dietary α-toc and δ-toc can result in their increased tissue concentrations in plasma, liver, and brain. Furthermore, co-supplementation of sesamin with high dosage δ-toc can increase δ-toc concentrations in plasma and liver to concentrations greater than those reached with high dosage dietary δ-toc alone. These findings will be valuable resources for future *in vivo* studies analyzing the effects of these two vitamin E isoforms on mammalian health as well as their potential for treatment of disease.

## Supplementary Files

Supplementary File 1: XLSX-Document (XLSX, 47 KB)
